# DNA topoisomerase IIα and mitosin expression predict meningioma recurrence better than histopathological grade and MIB-1 after initial surgery

**DOI:** 10.1371/journal.pone.0172316

**Published:** 2017-03-16

**Authors:** Theo L. Winther, Sverre H. Torp

**Affiliations:** 1 Departments of Laboratory Medicine, Children’s and Women’s Health, Norwegian University of Science and Technology (NTNU), Trondheim, Norway; 2 Pathology and Medical genetics, St. Olavs Hospital, Trondheim, Norway; Queen Mary Hospital, HONG KONG

## Abstract

**Background:**

The 2016 WHO histopathological grade or conventional biomarker MIB-1 is insufficient for predicting meningioma recurrence after initial treatment and alternative strategies are required. In this study, we investigated whether DNA topoisomerase IIα and/or mitosin expression can predict tumor recurrence with greater accuracy than conventional methods.

**Methods:**

The expression of MIB-1, topoisomerase IIα, and mitosin were determined as proliferation indices in tissue microarrays using immunohistochemistry. The accuracy of prognostication was assessed with receiver operating characteristic (ROC) analyses and standard survival analyses.

**Results:**

Expression of topoisomerase IIα and mitosin was significantly higher in recurrent meningioma than in non-recurrent meningioma (P ≤ 0.031), but no difference in MIB-1 expression was observed (P = 0.854). ROC analysis found topoisomerase IIα and mitosin expression to be the most reliable predictors of recurrence compared to WHO histopathological grade and MIB-1 expression. This result was supported by the multivariate survival analysis, in which mitosin expression was a significant predictor of recurrence-free survival (P < 0.001) and no association was found with histopathological grade or MIB-1 expression (P ≥ 0.158).

**Conclusions:**

The results suggest that topoisomerase IIα and mitosin improve prognostication of patients resected for meningioma. Tumors with higher topoisomerase IIα and/or mitosin expression have a higher risk of recurrence after initial treatment, and these patients may benefit from adjuvant treatment and closer radiological follow-up.

## Introduction

Meningioma is the most commonly reported primary brain tumor and accounts for more than one-third of such tumors [1]. According to the 2016 World Health Organization (WHO) histopathological classification for meningioma, most tumors are considered benign (Grade I) and are typically associated with good prognosis [2, 3]. However, a substantial proportion of tumors are associated with a greater likelihood of recurrence after surgery, or even death; such tumors correspond histopathologically to atypical and malignant meningiomas (Grade II and III, respectively) [2, 3].

Though surgery is the standard treatment, adjuvant radiation therapy has been shown to improve local control, disease-free survival, and overall survival for the more aggressive subsets of meningioma [4]. However, adjuvant radiation therapy includes a significant risk of treatment toxicity [4], and unnecessary exposure of patients with indolent tumors should be avoided.

The most important prognostic question regarding meningioma implies the prediction of recurrence after initial treatment [2, 3]. In this regard, the histopathological grading of meningioma is currently the most useful morphological tool for predicting prognosis [2, 3, 5]. The biggest drawback with this grading, however, is the substantial within-grade variation in recurrence risk among different grades [6–8], which complicates the decision-making process for treating these patients. Even among benign meningiomas, up to 20% are clinically aggressive and recur shortly after surgery [6, 9]. On the other hand, up to 71% and 50% of atypical and malign meningiomas, respectively, have indolent behavior with no recurrence [2, 3, 5]. These findings suggest that the histopathological grade is inadequate for predicting recurrence in patients resected for meningioma, and alternative methods are required for optimal decision-making regarding treatment of these patients.

Several additional and alternative methods have been suggested to predict recurrence more accurately [10–16]. Among these methods, cellular proliferation as measured by MIB-1 expression has been shown to correlate with the volume growth rate and is one of the most promising candidates in this setting [17–20]. However, the literature indicates several restrictions in applying this biomarker, including a considerable overlap of indices between recurrent and non-recurrent meningioma [19, 21]. Moreover, MIB-1 is associated with a heterogeneous staining pattern of varying intensity, making the interpretation and establishment of a definitive cutoff value that would translate between different laboratories difficult [22, 23].

Two cell cycle proteins, DNA topoisomerase IIα and mitosin, have shown potential in several tumors for overcoming these limitations and improving the prediction of meningioma recurrence [24–29]. Though the expression of mitosin has been described in all phases of the cell cycle except G0 and early G1 [30, 31], the expression of topoisomerase IIα is mostly restricted to G2 and M phase [32]. The expression of both proteins correlates with the expression of MIB-1, and they are also considered proliferation markers [23, 33]. However, a potential advantage of these biomarkers compared to MIB-1 is the homogenous and distinct immunoreactions described in different studies [22, 23], making the interpretation of topoisomerase IIα and mitosin expression more resistant to interlaboratory and interobserver variability.

Only a limited amount of previous research exists in this area. The results have been divided, with some demonstrating an association between the biomarkers and recurrence [33, 34] and others not finding an association [23]. Importantly, the statistical power has varied substantially from study to study, making it difficult to determine any definitive implications that can be adopted into clinical practice. In addition, most of these investigations were restricted to only evaluating the relationship between topoisomerase IIα or mitosin and recurrence, without investigating whether these biomarkers could complement or be surrogates for the histopathological grade. Application of these biomarkers in clinical practice as more accurate predictors of tumor recurrence could contribute to more personalized treatment decisions and improve the prognosis of patients suffering from meningioma.

The aim of this study was to investigate whether the expression of topoisomerase IIα and/or mitosin is associated with meningioma recurrence, and to compare the clinical usefulness of these biomarkers with the 2016 WHO histopathological grade and MIB-1 as predictors of prognosis.

## Materials and methods

### Patient selection

Patients were selected and clinical data collected as described previously [35, 36]. All patients who underwent meningioma surgery at St. Olavs Hospital, Trondheim University Hospital, Norway, over a 10-year period between January 1, 1991, and December 31, 2000, were retrospectively analyzed. Patients under the age of 18 years, with non-intracranial meningioma, or who received post-operative radiation immediately after surgery were excluded from the study. Six cases were also excluded due to an insufficient amount of tumor tissue for immunohistochemical analyses.

### Clinical data

Clinical data were collected from the hospitals’ medical records, including neurosurgery, radiology, and pathology records. Patient well-being before surgery was assessed based on clinical notes and classified according to the WHO guidelines for performance status. The extent of resection was defined according to the Simpson Resection Grade [37], which was assessed by the operating neurosurgeon or retrospectively based on the surgical notes when it was not explicitly stated. In both cases, the extent of resection was confirmed by post-operative magnetic resonance imaging. Gross total resection was defined as Simpson Grade I or II, and anything less than gross total resection was defined as subtotal resection.

Recurrence-free survival (RFS) was defined as the time from the date of the operation to the date radiological evidence of significant tumor growth was assessed by neuroradiologists at the hospital (i.e., magnetic resonance imaging, or computed tomography if magnetic resonance imaging was contraindicated).

Each meningioma case was reviewed independently by two researchers, one of whom was a senior neuropathologist (SHT), and classified according to the 2016 WHO histopathological grade [3]. For any discrepancies, cases were reviewed and consensus reached.

### Tissue microarray

Core extraction (1 mm diameter) was performed using an Alphelys Tissue Arrayer MiniCore® 3 (AH diagnostics) with the corresponding software TMA Designer2. Three cores were extracted from various histologically confirmed representative locations in each tumor to compensate for potential heterogeneity [38–41]. Whole-slide sections were included when an insufficient amount of tumor tissue was available for tissue microarray construction (n = 19).

### Immunohistochemistry

Following a standard procedure, immunohistochemistry was performed with anti-MIB-1 (clone MIB-1, dilution 1:50; Dako Denmark AS, Glostrup, Denmark), anti-topoisomerase IIα (clone KiS1, dilution 1:50, Dako Denmark AS, Glostrup, Denmark), and anti-mitosin (clone 14C10, dilution 1:10, Novus Biologicals, Cambridge, UK) using an automatic Dako Techmate 500. This procedure included pre-heating for 1 hour at 60°C and blocking endogenous peroxidase activity with 0.03% H_2_O_2_ for 10 minutes. Incubation was performed with PT Link Dako pre-treatment. Hematoxylin counterstaining was performed for all sections. Positive and negative controls from human tonsil tissue were included in each staining, and the primary antibodies were omitted from the negative controls. All antibodies were tested on several meningioma tissues with a broad range of dilutions before each staining to determine the optimal dilution for immunohistochemical assessment.

### Proliferation indices (PIs)

All meningioma cases were scored with a PI for each antibody based on the percentage of positive immunoreactive nuclei among 1000 tumor cell nuclei in the area of greatest proliferative activity (hot spots). The assessments were performed by two authors to ensure optimal reproducibility. Both investigators were blinded to the clinical data related to each case during the assessments.

### Statistical analysis

SPSS version 21.0 (SPSS Inc., Chicago, IL) was applied for statistical analyses. The Mann-Whitney U test was applied to evaluate the association between the proliferation markers and histopathological grade, in addition to the association with recurrence status. Receiver operator characteristic (ROC) was used to determine the optimal cutoff value based on maximization of the Youden index in order to discriminate between recurrent and non-recurrent meningioma, and the discriminatory power was tested with the chi-square test of association. The survival analyses were performed with univariate and multivariate Cox proportional hazards regression analyses and Kaplan-Meier curves. A P-value ≤0.05 was considered statistical significant.

### Ethics

This study was approved by the Regional Committee for Medical and Health Research Ethics Central Norway (project number 4.2006.947), and the study protocol adhered to the guidelines of the Helsinki Convention. Waiver of consent was given by the Regional Ethics Committee because patients were either deceased or severely disabled.

## Results

### Clinical data

A summary of the clinicopathological data according to histopathological grade is presented in Table 1. A total of 160 patients were included in the statistical analysis: 120 (75.0%) females and 40 (25.0%) males (ratio 3:1). The median age at surgery was 60 years (range 25–86 years). Gross total resection was achieved in 121 (75.6%) patients, and subtotal resection was performed in 39 (24.4%) patients. The RFS rate for the duration of follow-up was 77.7%. The median follow-up duration was 96 months (range 0–96 months).

**Table 1:** Clinical data according to 2016 WHO histopathological grade.

Clinicopathological feature	Grade I + II	Grade I	Grade II
Sex			
Female	120 (75.0)	90 (77.6)	30 (68.2)
Male	40 (25.0)	26 (22.4)	14 (31.8)
Median age (range), years	60 (25–86)	58 (27–84)	64 (25–86)
Simpson grade			
GTR	121 (75.6)	84 (72.4)	37 (84.1)
STR	39 (24.4)	32 (27.6)	7 (15.9)
WHO performance status			
0–1	134 (83.8)	97 (83.6)	37 (84.1)
2–5	26 (16.2)	19 (16.4)	7 (15.9)
Recurrence			
Yes	30 (18.8)	20 (17.2)	10 (22.7)
No	130 (81.2)	96 (82.8)	34 (77.3)

Data are presented as n (%) unless otherwise noted. GTR, gross total resection; STR, subtotal resection.

### Immunohistochemical staining

**Fig 1:**
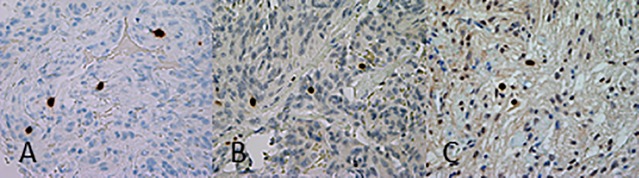
Immunostaining of proliferation markers. Examples of MIB-1 immunostaining (A), topoisomerase IIα immunostaining (B), and mitosin immunostaining (C) of meningioma (magnification 400×). The staining intensity was more homogenous and distinct for topoisomerase IIα and mitosin than for MIB-1.

Satisfactory immunohistochemical staining was achieved for all antibodies with clear distinction between positive immunoreactive tumor cell nuclei and non-reactive nuclei. Immunoreactivity was confined to the tumor cell nuclei for MIB-1 and topoisomerase IIα, but some cytoplasmic reaction was observed for mitosin. The staining intensity was more homogenous and distinct for topoisomerase IIα and mitosin than for MIB-1 (Fig 1).

### Association with histopathological grade and recurrence

The median PIs according to histopathological grade and recurrence status are presented in Table 2. For all proliferation markers, significantly higher expression was revealed in atypical meningioma compared to benign meningioma (P ≤ 0.028). However, a broad overlap of indices between tumor grades was observed for all markers.

**Table 2:** Differences in proliferation indices (PIs) between WHO grades and recurrent/non-recurrent meningioma.

	MIB-1 PI	Topoisomerase IIα	Mitosin
WHO grade			
Grade I	0.9 (0.0–5.3)	0.6 (0.0–4.9)	0.4 (0.0–2.5)
Grade II	1.8 (0.4–6.4)	0.9 (0.0–12.5)	0.8 (0.0–6.4)
P-value	< 0.001	0.028	< 0.001
Recurrent/non-recurrent			
Non-recurrent	1.2 (0.0–6.4)	0.6 (0.0–6.7)	0.5 (0.0–2.9)
Recurrent	1.1 (0.2–6.2)	1.0 (0.0–12.5)	0.6 (0.0–6.4)
P-value	0.854	0.031	0.018

PIs are defined as the percentage of positive immunoreactive nuclei among 1000 tumor nuclei. Data are presented as median (range). P-values were calculated using the Mann-Whitney U test.

Both topoisomerase IIα and mitosin were significantly associated with recurrence, with higher PIs in recurrent tumors than non-recurrent tumors (P ≤ 0.031, Table 2). No significant difference was found in MIB-1 expression (P = 0.854).

### Accuracy of predicting recurrence

Based on maximization of the Youden index in ROC analyses, the optimal cutoff values with respect to predicting recurrence for the MIB-1 PI, topoisomerase IIα PI, and mitosin PI were 3.0%, 1.0%, and 1.5%, respectively (Table 3). The cutoff values for the topoisomerase IIα PI and mitosin PI were significant predictors of recurrent tumors (P ≤ 0.039), whereas the cutoff value for the MIB-I PI and histopathological grade (Grade I vs. Grade II) did not reach significance (P ≥ 0.497). With the greatest area under the ROC curve, mitosin expression was the most accurate discriminator between recurrent and non-recurrent tumors.

**Table 3:** Receiver operator characteristics (ROC) analyses of cutoff values.

	Sensitivity (%)	Specificity (%)	Area under the curve	P-value*
WHO Grade	33.3	73.8	0.54	0.497
MIB-1 ≥ 3.0%	26.7	86.9	0.57	0.091
Topoisomerase IIα PI ≥ 1.0%	60.0	62.3	0.61	0.039
Mitosin PI ≥ 1.5%	33.3	91.5	0.62	< 0.001

*Chi-square test of association.

### Association with RFS

**Fig 2:**
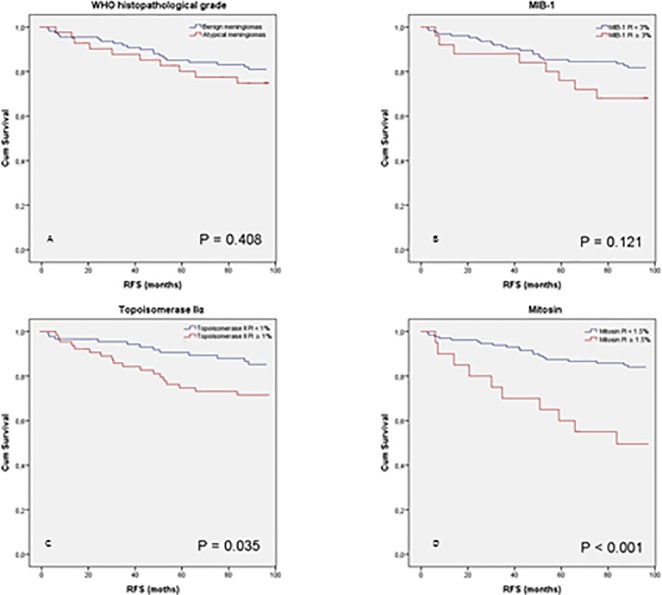
Kaplan-Meier curves and recurrence-free survival (RFS). Meningioma patients were stratified by optimal cutoff values revealed by receiver operator characteristics (ROC). Histopathological grade (A), MIB-1 PI (B), topoisomerase IIα (C), and mitosin (D). Topoisomerase IIα and mitosin expression were demonstrated to be predictors of RFS, but no association was found with histopathological grade or MIB-1.

Topoisomerase IIα and mitosin expression was associated with RFS (P ≤ 0.001, Table 4 and Fig 2). Comparing patients with topoisomerase IIα PI ≥ 1% and patients with topoisomerase IIα PI < 1% resulted in an increased hazard ratio of 3.12 (P = 0.001), and an increased hazard ratio of 4.00 was obtained for patients with mitosin PI ≥ 1.5% (P < 0.001). No association was found between RFS and MIB-1 expression or histopathological grade (P ≥ 0.127).

**Table 4:** Cox hazard univariate and multivariate survival analyses of proliferation assessment methods for recurrence-free survival.

	Hazard ratio (95% CI)	P-value
**Univariate analyses**		
WHO Grade	1.38 (0.64–2.94)	0.410
MIB-1 ≥ 3%	1.88 (0.84–4.22)	0.127
Topoisomerase II	2.15 (1.04–4.47)	0.040
Mitosin	4.00 (1.87–852)	< 0.001
**Multivariate analyses**		
*WHO Grade and clinical variables*		
Age	1.27 (0.61–2.62)	0.526
Simpson Grade	5.31 (2.52–11.19)	< 0.001
WHO performance status	1.07 (0.43–2.68)	0.879
WHO Grade	1.76 (0.81–3.85)	0.156
*MIB-1 and clinical variables*		
Age	1.00 (0.97–1.02)	0.739
Simpson Grade	4.79 (2.31–9.93)	< 0.001
WHO performance status	1.16 (0.47–2.85)	0.750
MIB-1 ≥ 3%	1.80 (0.80–4.05)	0.158
*Topoisomerase II and clinical variables*		
Age	1.15 (0.55–2.36)	0.706
Simpson Grade	4.82 (2.32–10.00)	< 0.001
WHO performance status	1.12 (0.45–2.73)	0.824
Topoisomerase	2.07 (0.99–4.31)	0.052
*Mitosin and clinical variables*		
Age	1.44 (0.69–3.03)	0.335
Simpson Grade	5.48 (2.60–11.54)	< 0.001
WHO performance status	0.99 (0.39–2.50)	0.984
Mitosin	4.80 (2.18–10.60)	< 0.001

The multivariate analyses were adjusted for clinically relevant variables. The date of surgery was used as a reference for the calculation of recurrence-free survival.

In multivariate survival analyses, the PIs and histopathological grade were adjusted for clinically known and relevant variables (age, Simpson Grade, and WHO performance status). Mitosin expression was the only significant predictor of RFS (hazard ratio = 4.80, P < 0.001), whereas the association with topoisomerase IIα expression was more uncertain (P = 0.052). The MIB-1 PI and histopathological grade remained insignificant predictors of RFS (P ≥ 0.158).

## Discussion

In the present study, we evaluated the expression of topoisomerase IIα and mitosin in a population-based series of human meningioma and analyzed their association with histopathological grade, recurrence status, and RFS. The prognostic values of these biomarkers were then compared with the recent 2016 WHO histopathological grade and the proliferation marker MIB-1. We found significantly higher expression of the three proliferation markers in atypical meningioma than benign meningioma. Increased expression of topoisomerase IIα and mitosin was also found in recurrent meningioma compared to non-recurrent meningioma, but no significant difference was found in MIB-1 expression. Furthermore, no significant association was found between histopathological grade or MIB-1 expression and recurrence rate in our series of meningiomas. Mitosin expression was revealed to be an independent predictor of RFS when adjusted for clinical variables. No associations between histopathological grade or MIB-1 expression and RFS were revealed in any of the survival analyses.

This is the first study to demonstrate an association between mitosin expression and histopathological grade. Konstantinidou et al. found no association between mitosin expression and histopathological grade in their series of meningiomas [33]. Their conclusion was based on a series of 47 tumors, 11 of which were classified as atypical meningioma. In contrast, we included 160 patients in our study, making this the largest investigation of mitosin expression in meningioma. Moreover, Konstantinidou et al. classified their tumors according to the 2000 WHO classification guidelines, whereas we reviewed and reclassified all tumors according to the guidelines of the 2016 WHO classification [2, 3]. This grading scheme includes brain invasion as a histological parameter of atypical meningioma, leading to more accurate determination of tumors with more aggressive behavior and less favorable outcome [3]. The inclusion of brain invasion as a parameter of aggressive histology also resulted in a higher frequency of atypical tumors. Poorer segregation between benign and atypical meningiomas may also have affected the results in Konstantinidou et al.’s study.

Two previous studies indicated an association between topoisomerase IIα expression and tumor aggressiveness [22, 34]. However, both of these studies classified the tumors according to the 2000 WHO classification. The study by Roessler et al. evaluated only the association between topoisomerase IIα expression and histopathological grade without investigating whether it is associated with recurrence status or RFS [22]. Furthermore, no survival analyses were performed in this study; therefore, it is difficult to draw any conclusions about the association with prognosis. In contrast, we demonstrated higher expression of topoisomerase IIα in recurrent meningioma compared to non-recurrent meningioma. In addition, we performed survival analyses to investigate the association with RFS. Both of these studies were also restricted to evaluating only the relationship between topoisomerase IIα and histopathological grade and MIB-1 expression without investigating whether these biomarkers could complement or be surrogates for these methods. In this study, we demonstrated that the expression of topoisomerase IIα is a more accurate predictor of recurrence compared to both the WHO classification and MIB-1 expression. This result indicates that application of this biomarker in clinical practice could contribute to more personalized treatment decisions and follow-up than what is possible today.

Cutoff values of 1.0% and 1.5% for topoisomerase IIα and mitosin expression, respectively, are consistent with earlier findings in the literature [33, 42]. However, with respect to the expression of MIB-1, a cutoff value of 3% is in agreement with several studies [43–47], though no general cutoff value has been established in the literature because a wide range of different values has been suggested in different studies (1% to 10%) [19]. This heterogeneity is likely the result of significant differences in techniques and interpretation between laboratories and observers [2]. The previous literature is also ambiguous on whether increased MIB-1 expression is associated with recurrence risk, with some studies demonstrating an association [20, 48] and others not [19, 21]. In our study, no significant relationship was found between MIB-1 expression and recurrence. In agreement with other studies, we also observed a heterogeneous staining pattern for MIB-1 with nuclear accentuation [22]. This observation may be explained by the variable expression of Ki-67 antigen throughout the cell cycle and, more importantly, the immunoreaction of this protein is weakened by prolonged fixation. Degradation of the antigen has been observed as soon as 1 week after cutting tumor tissue sections [34, 49, 50]. In contrast, the immunoreactions of topoisomerase IIα and mitosin were homogenous, distinct, and may be less prone to interobserver and interlaboratory variation.

One of the greatest drawbacks concerning the 2016 WHO classification is the substantial within-grade heterogeneity in recurrence risk [2, 7]. Thus, histopathological grading is insufficient to answer the prognostic question, which was also demonstrated in our series by the insignificant difference in recurrence rates between benign and atypical meningiomas and the poor ability to predict RFS.

Though most previous studies agreed on an association between topoisomerase IIα expression and meningioma recurrence, the literature is conflicting on whether the application of this biomarker has any advantage over MIB-1 [23, 42]. Some studies demonstrated a more sensitive prediction of recurrence with topoisomerase IIα [34], whereas others failed to prove any advantage over MIB-1 [22]. Based on the ROC analysis, this study showed that topoisomerase IIα expression is a more accurate prognosticator than MIB-1, revealing greater sensitivity and a greater area under the ROC curve. Utilizing the optimal cutoff values for both markers, topoisomerase IIα was also able to correctly identify more recurrent tumors than MIB-1 (18 vs. 8). In addition, considering the more distinct staining pattern with topoisomerase IIα, it would be a more useful biomarker in clinical practice.

We are only aware of one previous investigation of the expression of mitosin in meningioma, which only found a near significant association between mitosin expression and recurrence [33]. That study only included surgical patients with gross total resection, leading to the exclusion of tumors from surgically challenging locations and a number of recurrent tumors. Only seven tumors recurred in their series. In contrast, we included all surgically resected patients independent of location and extent of resection, and 30 tumors recurred in our series as a result. Though that study was limited to standard survival analyses, we extended our study with ROC analysis to compare the prognostic reliability to the histopathological grade and MIB-1 expression. To the best of our knowledge, this study is the first to make this comparison with the WHO histopathological grades of meningiomas, which has been adopted in most clinics as the major predictor of recurrence in combination with the extent of resection.

The expression of topoisomerase IIα and mitosin was demonstrated to more accurately predict recurrence in ROC analysis compared to both MIB-1 expression and histopathological grade. Mitosin was also the only significant predictor of RFS in multivariate survival analyses, as topoisomerase IIα did not reach significance. Therefore, patients with high mitosin and/or topoisomerase IIα expression may benefit from a more aggressive treatment approach, such as adjuvant radiation therapy with the possibility of re-resection, in addition to more frequent radiological follow-up. In contrast, for patients with low mitosin and/or topoisomerase IIα expression, periodic radiological follow-up is likely sufficient, with re-resection and/or radiation therapy in the case of recurrence. However, other risk factors must also be taken into consideration with regard to treatment decision-making, and the risk of surgery and adjuvant therapy must always be weighed against the possible benefits.

Our study has limitations inherent to the nature of retrospective studies and immunohistochemical analyses. Our study is also limited by the use of only one method to measure biomarker expression, as confirmation with a second method, such as Western blot or PCR, could have strengthened this study. However, PIs measured with immunohistochemical analyses, is more practical and thus more relevant as a routine method in the clinical practice. Furthermore, the assessment of proliferation on tissue microarray may be complicated by the heterogeneous expression pattern of the immunohistochemical markers. However, we tried to compensate for this potential limitation by extracting cores from three different histologically confirmed representative areas. Moreover, because construction of tissue microarray cylinders is a resource-intensive process, this method is not as relevant in daily clinical practice as it is for research purposes. In addition, recurrence does not always equal a worsening of symptoms or poor outcome, and the impact on the patients’ quality of life will vary. As with all immunohistochemical analyses, this study requires independent cohort studies for verification and optimization of the cutoff values and clinical usefulness.

In conclusion, we found higher expression of topoisomerase IIα and mitosin in recurrent meningioma compared to non-recurrent meningioma. Topoisomerase IIα and mitosin expression were identified as more accurate predictors of tumor recurrence than the 2016 WHO histopathological grade and MIB-1 expression. Furthermore, mitosin was the only significant predictor of RFS when controlling for other clinical factors. These findings suggest that the incorporation of topoisomerase IIα and/or mitosin into clinical practice may be useful and should be evaluated further in independent prospective studies.
